# Human cytomegalovirus promoting endothelial cell proliferation by targeting regulator of G-protein signaling 5 hypermethylation and downregulation

**DOI:** 10.1038/s41598-020-58680-6

**Published:** 2020-02-10

**Authors:** Xiaoni Zhang, Na Tang, Dongmei Xi, Qian Feng, Yongmin Liu, Lamei Wang, Yan Tang, Hua Zhong, Fang He

**Affiliations:** 1 Department of Pathophysiology/Key Laboratory of Education Ministry of Xinjiang Endemic and Ethnic Diseases, Medical College of Shihezi University, Shihezi, China; 2 Second Department of Emergency and critical care medicine, the First Affiliated Hospital of Medical College of Shihezi University, Shihezi, China; 3 Department of Geriatrics, the First Affiliated Hospital of Medical College of Shihezi University, Shihezi, China

**Keywords:** Mechanisms of disease, Cardiovascular biology

## Abstract

Interactions between human cytomegalovirus (HCMV) infection and environmental factors can increase susceptibility to essential hypertension (EH). Although endothelial dysfunction is the initial factor of EH, the epigenetic mechanisms through which HCMV infection induces endothelial cell dysfunction are poorly understood. Here, we evaluated whether HCMV regulated endothelial cell function and assessed the underlying mechanisms. Microarray analysis in human umbilical vein endothelial cells (HUVECs) treated with HCMV AD169 strain in the presence of hyperglycemia and hyperlipidemia revealed differential expression of genes involved in hypertension. Further analyses validated that the regulator of G-protein signaling 5 (*RGS5*) gene was downregulated in infected HUVECs and showed that HCMV infection promoted HUVEC proliferation, whereas hyperglycemia and hyperlipidemia inhibited HUVEC proliferation. Additionally, treatment with decitabine (DAC) and RGS5 reversed the effects of HCMV infection on HUVEC proliferation, but not triggered by hyperglycemia and hyperlipidemia. In summary, upregulation of RGS5 may be a promising treatment for preventing HCMV-induced hypertension.

## Introduction

Essential hypertension (EH) is a major risk factor of cardiovascular disease, involving endothelial dysfunction of large and small resistance arteries^1^. According to a previously performed study, EH is estimated to affect approximately 1.56 billion adults by 2025^2^. Despite this, the underlying cause and pathological mechanisms of EH have remained elusive. A number of previous studies revealed that hypertension is associated not only with genetic and environmental factors, but also with human cytomegalovirus (HCMV) infection, DNA methylation, and other epigenetic mechanisms^3^.

As reported previously, HCMV, which belongs to the β-herpes virus family, causes persistent infection in human^4,5^. HCMV accounts for 50–90% of the adult cases as a common opportunistic pathogen^6^. There is a growing evidence that cytomegalovirus infection may be associated with hypertension^7–10^. According to our previous researches, HCMV infection is related to essential hypertension in Kazakh men and Hans in Xinjiang, China, and HCMV antibody titers are associated with blood pressure and hypertension in Han men and women^11^. Studies demonstrated that HCMV can infect endothelial cells (ECs), macrophages and smooth muscle cells, which are all of great importance in the pathogenesis of vascular diseases^12,13^. HCMV infection is related to the pathogenesis of EH since it elicits vascular cells (e.g., ECs) dysfunction. In addition, mechanisms, such as dysfunction of ECs, inflammatory responses, CMV-specific T-cell responses to oxidative stress, and RAS activity indicate that HCMV infection is associated with EH.

Endothelial dysfunction is an early event in the pathophysiology of EH^14^. ECs are *in vivo* targets of HCMV infection and play a pivotal role in viral pathogenesis^15–17^. Besides, ECs are the first site of HCMV infection, which are a potential virus reservoir for virus persistence and spread^18^. HCMV facilitates developmental processes of vascular diseases, including initial damage and subsequent inflammation, and increases proliferation of ECs^19^. A recently conducted research demonstrated that HCMV infection may influence the proliferation of ECs through mammalian target of rapamycin (mTOR) signaling pathway^20^. Additionally, HCMV-mir-UL112 affects proliferation and growth of ECs^21^. However, the underlying mechanisms, in which HCMV induces EH, particularly the mechanisms of HCMV infection regulating endothelial dysfunction, have remained obscure.

DNA methylation plays significant regulatory roles in both normal and pathological cellular processes^22–25^. Abnormal DNA methylation patterns are typically observed in human diseases, including cancer^26,27^, autoimmune disease^28^, and cardiovascular diseases, such as hypertension^23,29^. Furthermore, the results of a previous study indicated that HCMV infection in human may cause changes in genomic DNA methylation levels^30^. Moreover, DNA methylation has been shown to influence cell susceptibility to HCMV infection^31^. However, it needs to be clarified whether HCMV infection is involved in the development of hypertension through changes in DNA methylation levels.

Hence, in the present study, we attempted to investigate whether HCMV infection interacted with environmental factors to participate in the development of EH through endothelial dysfunction and gene DNA methylation.

## Results

### mRNA expression profiles

**Figure 1:**
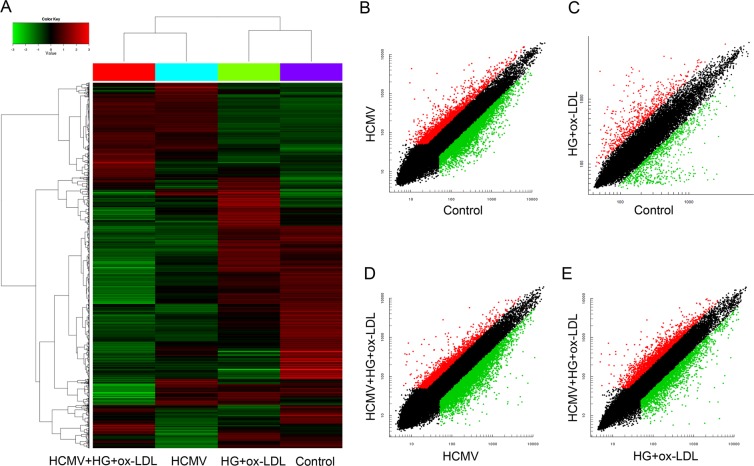
mRNA microarray expression data from four groups (HCMV-infected group (HCMV), high glucose (HG) and oxidized low-density lipoprotein (ox-LDL) group (HG + ox-LDL), HCMV-infected plus HG and ox-LDL group (HCMV + HG + ox-LDL), control). (**A**) Heat map of significantly-changed, the expression of mRNA is hierarchically clustered on the y axis, and four groups are hierarchically clustered on the x axis. The relative mRNA expression is depicted according to the color scale shown on the Left. Red indicates upregulation; green, downregulation. (**B**) The scatter plot of signal value between the groups of control and HCMV. (**C**) Control compared with HG + ox-LDL. (**D**) HCMV + HG + ox-LDL compared with HCMV. (**E**) HCMV + HG + ox-LDL compared with HG + ox-LDL. Red indicates upregulation; green, downregulation.

To determine differential mRNA expression profiles in the context of HCMV infection plus environmental risk factors, we screened mRNA expressions in four groups of HUVECs. The mRNA levels were compared in each group, as illustrated in the heat map (Fig. 1A). Scatter plots showed that there were 2,335 upregulated and 3,275 downregulated genes. After separating signal from noise and performing the Student’s t-test, significant differences in mRNA expressions of up to 2-fold were noted (*P* < 0.05; Fig. 1B). Besides, 3290 mRNAs were significantly differentially expressed (*P* < 0.05) when HCMV-infected cells were compared with HCMV-infected cells treated by HG and ox-LDL (*P* < 0.05; Fig. 1C). Additionally, 371 mRNAs were upregulated, whereas 545 mRNAs were significantly downregulated in HCMV-infected HUVECs treated by HG and ox-LDL compared with those in control cells (*P* < 0.05; Fig. 1D). Of the 8,579 mRNAs significantly altered in comparison with HUVECs treated by HG and ox-LDL with or without HCMV infection, 2,472 genes were upregulated, whereas 3,304 were downregulated (*P* < 0.05; Fig. 1E). As shown in Table 1, regulation of gene expressions in endothelial dysfunction may be influenced by HCMV infection.

**Table 1:** The number of differentially expressed genes between different groups.

Group	upregulation	downregulation
Control vs HG + ox-LDL	371	545
Control vs HCMV	2335	3275
HCMV vs HG + ox-LDL + HCMV	1569	1721
HG + ox-LDL vs HG + ox-LDL + HCMV	2472	3304

Note. Control vs HG + ox-LDL: control compare with high glucose (HG) and oxidized low-density lipoprotein (ox-LDL);

Control vs HCMV: control compare with HCMV- infected;

HCMV vs HG + ox-LDL + HCMV: HCMV- infected compare with HCMV-infected plus HG and ox-LDL;

HG + ox-LDL vs HG + ox-LDL + HCMV: high glucose and lipid compare with HCMV-infected plus HG and ox-LDL.

### GO (gene oncology) enrichment analysis of DEGs (differentially expressed genes) and enrichment analysis of pathways

**Figure 2:**
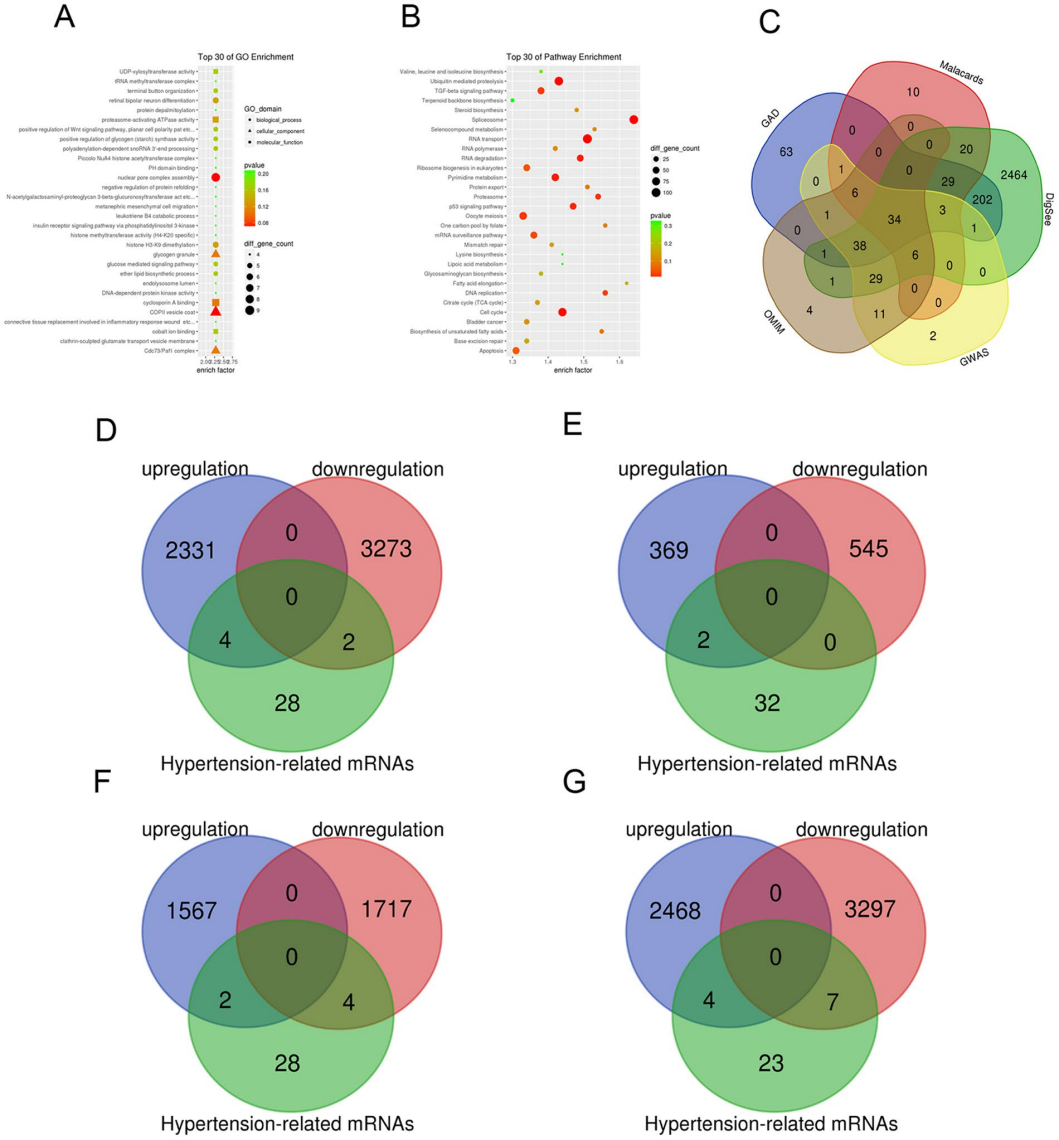
Hypertension related target gene screening. (**A**) The significant GO terms that conformed to a *P* < 0.05 were screened. (**B**) Fisher’s exact test was used to select the significant pathway, identified by a *P* < 0.05. GO, Gene ontology; BP, Biological process; CC, Cellular component; MF, Molecular function. (**C**) Venn diagrams of DGEs mRNA in the hypertension related gene set, Online Mendelian Inheritance in Man (OMIM), Genetic Association Database (GAD), the Human Gene Database (MalaCards), Disease Gene Search with Evidence Sentences (Digsee), genome-wide association studies (GWAS). (**D**) Venn diagrams of significantly upregulated or downregulated mRNAs in the hypertension related gene control compared with HCMV. (**E**) Venn diagrams of significantly upregulated or downregulated mRNAs in the hypertension related gene Control compared with HG + ox-LDL. (**F**) Venn diagrams of significantly upregulated or downregulated mRNAs in the hypertensionrelated gene HCMV + HG + ox-LDL compared with HCMV. (**G**) Venn diagrams of significantly upregulated or downregulated mRNAs in the hypertensionrelated gene HCMV + HG + ox-LDL compared with HG + ox-LDL.

To elucidate endothelial dysfunction-linked biological processes affected by HCMV infection, a GO functional enrichment analysis was conducted on DEGs. The majority of GO terms were related to biological processes, e.g., protein and nucleic acid metabolism, DNA replication, and regulation of oxidative stress (Fig. 2A). The pathway annotation of differentially expressed genes was performed and the obtained DEGs were all involved in pathway terms using the KEGG database. The DEGs were mainly enriched in pathways, for instance, cell apoptosis, cell cycle, DNA duplication, adhesion factors, and actin cytoskeleton regulation (Fig. 2B).

Two major objectives of the present research were as follows: 1) to investigate the correlation degree between disease and genes, and 2) to clarify genes’ role in disease. Thus, according to disease enrichment obtained from our results, the most obvious diseases were colorectal cancer, vascular disease, and cardiac hypertrophy. Therefore, five different databases were selected to evaluate the enrichment of EH (Fig. 2C). The data indicated that several DEGs, including prostaglandin I2 (prostacyclin) synthase (*PTGIS*), *RGS5*, selectin E (*SELE*), endothelin converting enzyme (*ECE*), and ATPase, Na^+^/K^+^ transporting, and beta 1 polypeptide (*ATP1B1*), were highly associated with hypertensive disease (Fig. 2D–G and Table 2). It has been indicated that HCMV infection may be related to hypertension in the Kazakh Chinese population in Xinjiang province (China) through serum angiotensin-converting enzyme (sACE) hypomethylation and 11-d hydroxysteroid dehydrogenase 2(HSD11β2) hypermethylation^30^. Thus, we attempted to further identify the seven candidate genes related to hypertension.

**Table 2:** Enrichment of hypertension by different databases.

Group	upregulation	downregulation
Control vs HCMV	ADRB3 ACE PTGIS ERAP1	RGS5 NOS3
Control vs HG + ox-LDL	ATP1B1 PTGIS	—
HCMV vs HG + ox-LDL + HCMV	ATP1B1 RGS5	CYBA ERAP1 ADRB2 PTGIS
HG + ox-LDL vs HG + ox-LDL + HCMV	GRK4 ADRB3 ACE ATP1B1	RGS5 PTGIS NEDD4L CYBA ERAP1 ADRB2 NPR2

Note. Adrenoceptor beta 3(ADRB3); Angiotensin-converting enzyme(ACE); Prostaglandin I2 (prostacyclin) synthase (PTGIS); Endoplasmic reticulum aminopeptidase 1(ERAP1); Regulator of G-protein signaling 5(RGS5), Nitric oxide synthase 3(NOS3); ATPase, Na+/K+ transporting, beta 1 polypeptide(ATP1B1); Cytochrome b-245, alpha polypeptide(CYBA); Endoplasmic reticulum aminopeptidase 1(ERAP1); Adrenoceptor beta 2, surface (ADRB2); G protein-coupled receptor kinase 4(GRK4); Neural precursor cell expressed, developmentally down-regulated 4-like, E3 ubiquitin protein ligase (NEDD4L); endoplasmic reticulum aminopeptidase 1 (ERAP1); Adrenoceptor beta 2, surface (ADRB2); Selectin E (SELE); Natriuretic peptide receptor 2(NPR2).

### Validation of microarray results using RT-qPCR(quantitative reverse transcription polymerase chain reaction)

**Figure 3:**
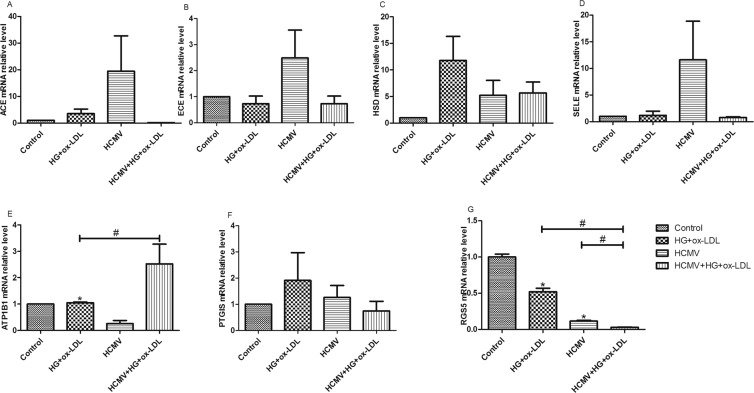
mRNAs were validated in an expanded cohort using RT-qPCR with mRNA-specific primers, after exclusion of outliers. The *p*-values were calculated by two-tailed unpaired Student’s t-tests. *P < 0.05 vs control; ^#^*P* < 0.05 vs HCMV or HG + ox-LDL.

To further evaluate significance of the seven identified DEGs, we examined mRNA expressions using RT-qPCR. The mRNA expressions of candidate genes among different groups are revealed by Fig. 3. It was demonstrated that *ATP1B1* expressions in uninfected HUVECs treated by HG and ox-LDL were higher than those in the control group, while those were lower than those in HCMV-infected HUVECs treated by HG and ox-LDL (Fig. 3E). Importantly, no significant differences in the expression levels of *ACE*, *ECE*, *HSD*, *SELE*, and *PTGIS* were observed in RT-qPCR analysis (Fig. 3A–D,F). The *RGS5* gene expression level was reduced in the HCMV-infected group and in the uninfected group treated by HG and ox-LDL compared to the control; the decreased expression level of *RGS5* was more substantial in HCMV-infected HUVECs treated by HG and ox-LDL than that of the other two experimental groups (Fig. 3G). These results above were in agreement with those obtained from the microarray chip analysis. Therefore, *RGS5* was selected as a target gene to investigate its role in the function of ECs, involving the interaction of HCMV infection with environmental risk factors.

### HCMV infection promoted EC proliferation

**Figure 4:**
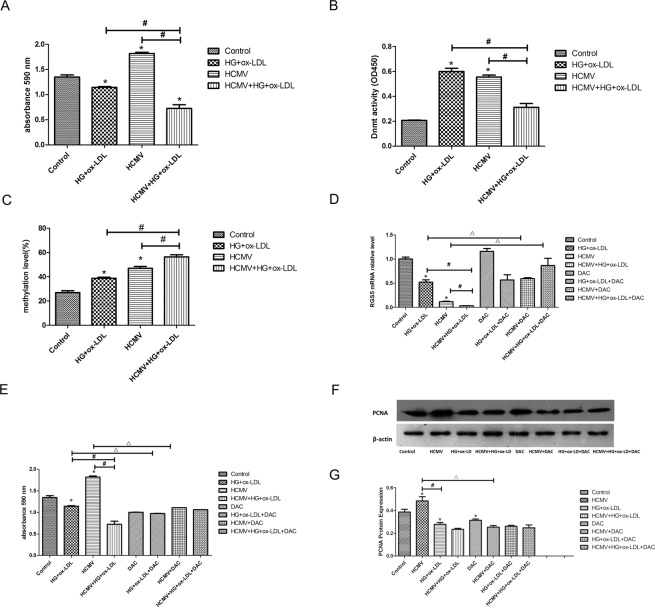
Interaction of HCMV and environmental risk factors on endothelial cell proliferation by DNA methylation. (**A**) Changes in cell proliferation function in each treatment group by CCK-8 analysis. (**B**) Detection of methyltransferase activity in different treatment groups. (**C**) The methylation levels of CpG sites in RGS5 promoter regions from the four groups. (**D**) Effect of DAC on the expression of RGS5mRNA.*P < 0.05 vs control; ^#^*P* < 0.05 HCMV+HG + ox-LDL vs HCMV or HG + ox-LDL. (**E**) Effect of DAC on cell proliferation. (**F,G**) Effect of DAC on the expression of PCNA protein. Data are expressed as means ± standard deviations. *P < 0.05 vs control; ^#^*P* < 0.05 HCMV + HG + ox-LDL vs HCMV or HG + ox-LDL. ^Δ^*P* < 0.05 HCMV vs HCMV + DAC, HG + ox-LDL vs HG + ox−LDL + DAC. Student’s t-test.

Next, CCK-8 assay was undertaken to determine the role of HCMV infection in HUVECs proliferation. As illustrated in Fig. 4A, HCMV infection significantly increased the proliferation of HUVECs compared with the control (*P* < 0.05). In contrast, the proliferation of HUVECs treated by HG and ox-LDL was reduced (*P* < 0.05). Moreover, for HCMV-infected HUVECs treated by HG and ox-LDL, the proliferation of ECs was further decreased compared with the HCMV-infected group or the HG and ox-LDL-treated group (*P* < 0.05). The facts above indicated that HCMV infection promoted the proliferation of ECs, while proliferation of ECs was inhibited by HG and ox-LDL treatment, which enhanced EC proliferation caused by HCMV infection, and that could be blocked by HG and ox-LDL treatment.

### HCMV infection significantly increased activity of DNMT in ECs

To assess the underlying mechanisms, in which the interaction between HCMV infection and environmental risk factors influenced endothelial dysfunction, we initially tested the activity of DNMT in the four treatment groups. The results showed that the activity of DNMT in HCMV-infected or HG and ox-LDL-treated HUVECs was increased compared to the control (*P* < 0.05). Additionally, the activity of DNMT was decreased in HCMV-infected HUVECs treated by HG and ox-LDL compared with the other two experimental groups (*P* < 0.05; Fig. 4B).

### DNA hypermethylation was involved in HCMV-induced *RGS5* downregulation

It was revealed that HCMV infection promoted the activity of DNMT. Thus, we further investigated whether HCMV infection induced *RGS5* downregulation via DNA hypermethylation. Besides, RGS5 methylation levels were significantly increased in both HCMV-infected HUVECs and HUVECs treated by HG and ox-LDL compared to the control (*P* < 0.05). Additionally, the increase in *RGS5* methylation in HCMV-infected HUVECs treated by HG and ox-LDL was further amplified compared with HCMV-infected or HG and ox-LDL-treated cells (Fig. 4C).

To further indicate whether DNA hypermethylation was associated with RGS5 downregulation, HUVECs were incubated in the presence of decitabine (DAC). It was disclosed that RGS5 mRNA expressions in HCMV-infected or HG and ox-LDL-treated HUVECs were lower compared to the control (*P* < 0.05). The combined effects of the two groups further decreased *RGS5* mRNA expressions (*P* < 0.05), which is consistent with the above-mentioned results of *RGS5* methylation, indicating that HCMV infection and HG and ox-LDL treatment could synergistically reduce *RGS5* mRNA expressions. Moreover, the *RGS5* mRNA expression in HCMV-infected or HG and ox-LDL-treated HUVECs was increased upon the addition of DAC (*P* < 0.05; Fig. 4D; the four sets of DAC-free data were identical to Fig. 3G, and this figure verified mRNA expression levels of RGS5; besides, Fig. 4D illustrated the effects of DAC treatment on mRNA expression levels of RGS5). These outcomes demonstrated that HCMV infection, together with environmental risk factors, such as high levels of glucose and fat, regulated the expression level of RGS5 through methylation, thereby influencing the function of ECs.

### HCMV infection-induced EC proliferation was regulated by DNA methylation

In the present research, we focused on the effects of DNA methylation on the regulation of EC dysfunction in the context of interactions between HCMV infection and environmental risk factors using CCK-8 assay. Upon treatment with DAC, the enhanced proliferation of ECs induced by HCMV infection was reversed, whereas HG and ox-LDL treatment did not affect the inhibition of proliferation of ECs (*P* < 0.05), as depicted by Fig. 4E (the four sets of DAC-free data were identical to Fig. 4A so as to more accurately compare effects of DAC treatment on proliferation of ECs). The expression level of PCNA in HCMV-infected cells was significantly higher (*P* < 0.05), whereas that level in HUVECs treated with HG and ox-LDL was notably lower compared to the control group (*P* < 0.05). Additionally, the expression level of PCNA was remarkably lower in HCMV-infected HUVECs treated with HG and ox-LDL than in uninfected HUVECs treated with HG and ox-LDL (*P* < 0.05). These findings indicated that HCMV infection promoted the expression level of PCNA, whereas HG and ox-LDL inhibited the expression level of PCNA. Notably, the expression level of PCNA was lower in DAC-treated group than that in control group (*P* < 0.05), iindicating that DNMT inhibitors could reverse the expression level of PCNA. Moreover, the expression level of PCNA in HCMV-infected HUVECs treated with DAC was lower than that in untreated HCMV-infected HUVECs (*P* < 0.05; Fig. 4F,G). Thus, it could be concluded that DNMT inhibitors could reverse the high expression of PCNA induced by HCMV infection. As previously described, our findings demonstrated that HCMV infection facilitated proliferation of ECs by regulating the RGS5 expression level through methylation.

### Overexpression of RGS5 reversed EC proliferation triggered by HCMV infection

**Figure 5:**
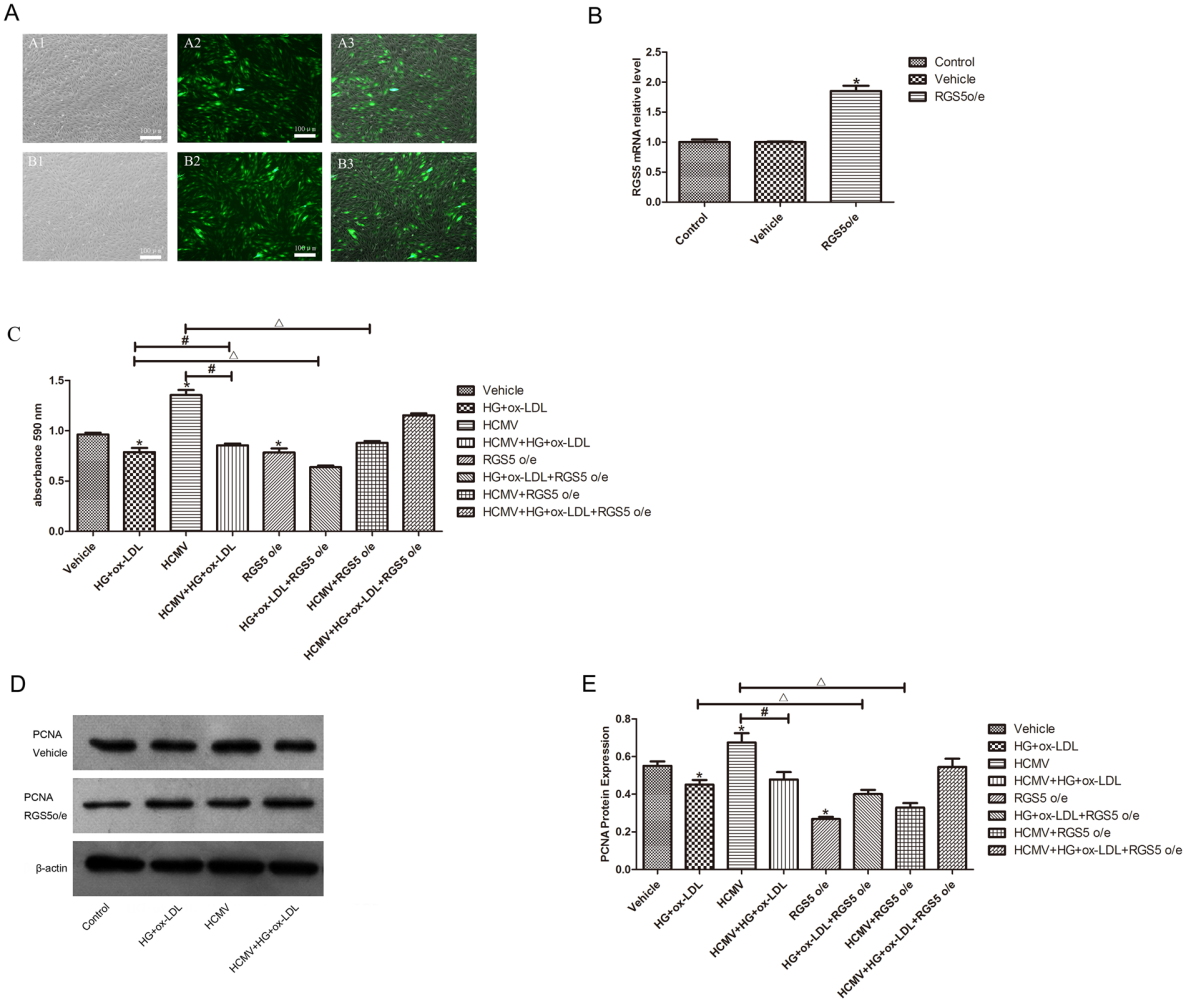
HCMV infected promotes endothelial cell proliferation through low expression of RGS5. (**A**) Green fluorescence was observed in both the RGS5-transfected and GFP-positive control groups, while that was not observed in the untransfected group. (**B**) RGS5 expression levels in HUVECs were detected by real-time PCR. RGS5 expression signifcantly increased after RGS5o/e adenovirus infected. (**C**) RGS5o/e reversible cell proliferation induced by HCMV infected by CCK-8 analysis. (**D,E**) RGS5o/e reduces the increase in PCNA induced by HCMV infected. RGS5o/e(RGS5-overexpression vector). *P < 0.05 vs control; ^#^*P* < 0.05 HCMV + HG + ox-LDL vs HCMV or HG + ox-LDL. ^Δ^*P* < 0.05 HCMV vs HCMV + RGS5 was considered statistically signifcant. Student’s t-test.

We also assessed the effects of overexpression of RGS5 on proliferation of ECs. Green fluorescence was observed in both the RGS5-transfected and GFP-positive control groups, while that was not observed in the untransfected group (Fig. 5A), demonstrating that the adenovirus transfection was successful, with a transfection rate of more than 90%. Additionally, RT-qPCR analysis further confirmed the successful construction and transfection of the RGS5-overexpression vector (*P* < 0.05; Fig. 5B).

Cell proliferation was measured using CCK-8 assay, and it was uncovered that proliferation of ECs in the RGS5-overexpression group was lower than that in the vehicle group (*P* < 0.05). Notably, proliferation of ECs in HCMV-infected cells overexpressing RGS5 was lower compared to HCMV-infected cells without overexpression of RGS5 (*P* < 0.05; Fig. 5C). To further elucidate the effects of RGS5 on proliferation of ECs induced by HCMV infection, the expression level of PCNA was measured after overexpression of RGS5. As depicted in Fig. 5D,E, overexpression of RGS5 decreased the expression level of PCNA protein, suggesting that proliferation of ECs could be induced by HCMV infection via hypermethylation and downregulation of *RGS5*.

## Discussion

In our previous research, it was indicated that HCMV infection was associated with hypertension in Kazakh and Han Chinese populations, and interactions with environmental factors, e.g. HG and high fat, could increase susceptibility to hypertension^11^. Our study first found that *RGS5*, a negative regulator of G protein-coupled receptors (GPCRs), was a target gene for dysfunction of ECs caused by HCMV infection, while that was not triggered by HG or ox-LDL. Furthermore, it was demonstrated that HCMV infection downregulated *RGS5* by DNA hypermethylation, resulting in increased proliferation of ECs. The evidence can be described as follows: First, HCMV infection or hyperglycemia and hyperlipidemia have the same downregulation effect on RGS5 caused by DNA hypermethylation, and they have synergistic influence, while opposite effects on the proliferation of ECs were noted. Second, methyltransferase inhibitor could only reverse the enhanced proliferation of ECs mediated by HCMV infection, whereas accelerate the inhibitory influences of hyperglycemia and hyperlipidemia on the proliferation of ECs. In addition, the overexpression of RGS5 reversed the regulatory effect of HCMV on proliferation of ECs, while did not affect the regulation of HG and high fat on proliferation of ECs.

In the current research, it was revealed that there were several DEGs in the HCMV-infected group compared to the control. Among these DEGs, more than 2000 genes were upregulated, and 3275 genes were downregulated. The FBJ murine osteosarcoma viral oncogene homolog gene showed the highest upregulation with a FC of up to 57, indicating that the mentioned gene was remarkably influenced by HCMV infection. However, HUVECs pretreated with HG and ox-LDL showed less induction of DEGs, with 371 upregulated genes and 545 downregulated genes, suggesting that the transcriptional features of cells were altered under different environmental conditions. When the relationships between DEGs in the HCMV-infected group and HG- and ox-LDL-treated group were compared, we found that the DEAD box helicase 3, Y-linked gene was the main DEG with a FC of almost 100. This gene was found to be involved in ATPase metabolism, intracellular electron conversion, and other biological processes. Thus, these data indicated that ECs induced innate immune responses to prevent infection by viruses. Additionally, HCMV infection resulted in substantial numbers of DGEs, and the majority of them were downregulated. This indicated that the reproductive mechanism of viruses, e.g. by borrowing the replication machinery of the host cell to force replication of the genetic material of the virus itself, led to the downregulation of the majority of DEGs in the ECs.

In enrichment analysis, DEGs were mainly involved in apoptosis, cell cycle, DNA replication, adhesion factors, and actin cytoskeleton regulation. According to the results of GO and functional enrichment analysis, the DEGs induced by infection were mainly enriched in protein metabolism, nucleic acid metabolism, and regulation of oxidative stress. These results demonstrated that HCMV infection induced oxidative stress in ECs, leading to tissue damage. A previous study uncovered that HCMV infection increased the production of reactive oxygen species (ROS) in ECs, leading to cellular oxidative stress. Upon generation of ROS, inflammatory cell infiltration could be increased by enhancing cell permeability, thereby influencing cell proliferation, activating multiple signal transduction pathways, mediating migration and differentiation of monocytes and secretion of cytokines by macrophages, as well as promoting the occurrence and development of hypertension^32^. This is consistent with our previous results that patients with EH, particularly those with HCMV infection, exhibited a remarkable increase in 8-hydroxy-2-deoxyguanosine levels, which may have contributed to development of EH in the Kazakh Chinese population^30^. Further screening of DEGs based on disease enrichment and previous studies showed that only the expression level of *RGS5* was altered in RT-qPCR analysis. Thus, we concentrated on this gene in the subsequent analyses.

Moreover, RGS5 is an important modulator of signal transduction in the cardiovascular system and is highly expressed in the aorta, heart, skeletal muscle in smooth muscle cells (SMCs), and ECs in cardiovascular tissues^33–35^. Moreover, RGS5 plays substantial roles in remodeling blood vessels and regulating blood pressure^36–38^. A study demonstrated that RGS5 may be a candidate target gene for the treatment of human and animal hypertension. Indeed, RGS5 genetic polymorphisms have been shown to be associated with human hypertension^39^. Additionally, the expression level of RGS5 in the vascular smooth muscle and intima was markedly reduced in spontaneously hypertensive rats and adrenocorticotropic hormone- and Ang-II-induced hypertensive rats, while the blood pressure level was increased in RGS5-knockout mice^37,40,41^. Moreover, RGS5 might also be involved in the regulation of blood pressure by modulating Na^+^ transport in renal epithelial cells, G renal epit AngII type 1 receptor signaling, protein kinase C, mitogen-activated protein kinase (MAPK)/extracellular signal-regulated kinase (ERK), RhoA kinase activation-mediated increases in vascular resistance and vascular remodeling, and downregulation of peroxisome proliferator-activated receptor β-induced oxidative stress and inflammatory responses^37,42–44^. However, it is still elusive whether RGS5 can induce dysfunction of ECs in the context of HCMV infection. It has been reported that HCMV infection promotes proliferation of ECs^45^. Similarly, our study found that HCMV infection stimulated the proliferation of ECs, whereas HG and ox-LDL inhibited proliferation of ECs. In addition, the interactions between these factors further reduced proliferation of ECs. ECs grown under hyperglycemic conditions have been shown to exhibit decreased proliferation and fibrinolytic potential, in addition to increased apoptosis^46,47^. Additionally, a high concentration of ox-LDL decreases migration and proliferation of ECs^48^. Li *et al*. found that hyperglycemia and hyperlipidemia enhanced the expression levels of ox-LDL receptors (e.g., LOX-1) in ECs^49–51^; this can induce apoptosis, suggesting that the effects of HG and lipids on inhibiting proliferation of ECs may be related to the induction of apoptosis of ECs.

It is noteworthy that HCMV infection induces development of a series of cardiovascular diseases^52^, and ECs can serve as host cells for HCMV infection^53^. However, the molecular mechanisms, in which HCMV infection regulates proliferation of ECs, leading to endothelial dysfunction and causing vascular diseases, have remained obscure yet. Our study indicated that the activity of DNMT and DNA methylation levels were increased in ECs after HCMV infection or HG and ox-LDL treatment. Importantly, global DNA hypermethylation and gene-specific methylation have been implicated in cardiovascular diseases^54,55^. Besides, HCMV infection has been reported to inhibit DNA methylation in host cells, which in turn affects cell susceptibility to HCMV infection^31^. HCMV infection decreases the methylation of the human aquaporin-1 gene in the submandibular gland of mice, resulting in the occurrence of salivary gland inflammation^56^. As reported previously, HCMV infection induces *sACE* hypomethylation and *HSD11β-2* hypermethylation in peripheral blood mononuclear cells, which could be involved in the occurrence of EH in the Xinjiang Kazakh population^57^. Notably, in the present research, we found that DAC reversed the low expression level of RGS5 caused by HCMV infection. Furthermore, DAC reversed proliferation of ECs induced by HCMV infection, while further aggravated the inhibition of ECs by hyperglycemia and hyperlipidemia. Based on these findings, we suggest that HMCV infection may induce proliferation of ECs by reducing the expression level of *RGS5* through DNA methylation.

Animal experiments have confirmed that mRNA levels of *RGS5* decrease in vascular SMCs in atherosclerotic lesions^58^, and loss of *RGS5* results in profound hypertension^37^. Downregulation of RGS5 has been identified in bypass graft neo-intima, atherosclerotic arteries, and hypertension^42,59,60^. Consistent with these findings, we found that RGS5 overexpression reversed the proliferation of ECs induced by HCMV infection, whereas did not influence the effects of HG or ox-LDL on proliferation of ECs. Several previous studies reported that ox-LDL suppressed cell proliferation though inhibiting expression levels of basic fibroblast growth factor-5 (bFGF-5) or nuclear translocation of cell-cycle proteins^61^. Ox-LDL also suppresses vascular endothelial growth factor-induced EC migration via its inhibitory effect on the AKT/endothelial nitric oxide synthase pathway^62^. In addition, HG regulates bFGF-2 to influence proliferation of ECs through changes in permeability of ECs^63^. The mechanisms, in which hyperglycemia and hyperlipidemia induce proliferation of ECs, are complex and may involve various metabolic abnormalities; however, a low expression level of *RGS5* owing to hypermethylation may not be one of these mechanisms. Overall, our data demonstrated that HCMV infection could reduce the expression level of *RGS5* by promoting hypermethylation, thereby increasing the proliferation of ECs.

The current research contains several limitations. First, further studies need to be conducted to verify our *in vitro* results. Indeed, because HCMV infection is a complicated process involving the interactions of virus, host, and host cell factor-1, further analyses are essential. Additionally, to facilitate the application of these findings to human disease, further studies in human and animal models are required, and the exact mechanisms and possible therapeutic applications should be meticulously evaluated. Second, we are entering a new era of understanding how genomes interact with the environment to influence the pathogenesis of diseases. A new evidence showed that epigenetic and genetic factors are essential for regulation and maintenance of blood pressure, and there are complex interactions among genetic and environmental factors, thereby influencing the risk of EH. In the current experiments, we, for the first time, observed that the mechanisms of DNA methylation in the occurrence and development of EC dysfunction were related to hypertension. HCMV infection can induce differential expressions of host genes, and lead to changes in DNA methylation levels in the host. The results of the current research demonstrated that HCMV infection promoted proliferation of ECs by downregulating the expression level of *RGS5* via DNA hypermethylation. To our knowledge, we first demonstrated that interactions among HCMV infection, environmental risk factors, and DNA methylation may lead to EC dysfunction, and thus, participate in the occurrence and development of EH.

In summary, in this study, we provided evidence demonstrating that RGS5 was an important negative regulator of the proliferation of ECs following HCMV infection. Mechanistically, we showed that DNA hypermethylation promoted the proliferation of ECs by RGS5 downregulation. Our findings provided a basis for exploring methods for CMV treatment that could also prevent EH, either directly with antivirals or by modulation of RGS5 induced by the virus. The main toxicity of systemic ganciclovir is neutropenia, and although foscarnet is an effective drug for overcoming this limitation of ganciclovir, its main side effects include nephrotoxicity and electrolyte imbalance^64^. Thus, it is urgent to find an effective vaccine and new antiviral intervention strategies to mitigate the deficiencies and toxicities of existing antiviral drugs^65,66^. Accordingly, RGS5 may represent a promising therapeutic target to prevent EH induced by HCMV infection.

## Materials and Methods

### Cell culture and viral titers

Human umbilical cords (from patients with normal body weight) were obtained after full-term normal deliveries under protocols approved by the Ethics Committee of Obstetrics and Gynecology of an affiliated hospital of Shihezi University, and informed consent was obtained from all the patients. Human umbilical vein endothelial cells (HUVECs) were isolated and grown in endothelial cell medium (ECM; Life Technologies, Carlsbad, CA, America). HUVECs were cultured in ECM supplemented with fetal bovine serum (FBS; 10%) (HyClone, Logan, UT, America), streptomycin (0.1 mg/mL) and penicillin (100 IU/mL). Human embryonic lung fibroblast cells (MRC-5 cells) were purchased from Wuhan Institute of Virology (Wuhan, China), and were maintained in Dulbecco’s modified Eagle’s medium (DMEM; Life Technologies, Carlsbad, CA, America) supplemented with FBS (10%), penicillin (100 IU/mL), and streptomycin (0.1 mg/mL). All cells were cultured at 37 °C in presence of 5% CO_2_.

The laboratory-adapted strain of HCMV AD169 obtained from Wuhan Institute of Virology (Wuhan, China) was propagated in confluent monolayers of MRC-5 cells in DMEM supplemented with FBS (10%), penicillin (100 IU/mL), and streptomycin. Supernatants of the infected MRC-5 cells displaying 90–100% cytopathic effects were collected, and centrifuged, which were then stored under −80 °C until the next analysis. Plaque assays were performed to determine Viral stock titers, which were calculated as 10^5^ PFU/ml.

### HCMV infection

HUVECs were plated in 12-well plates and cultured in ECM until cells reached 70–80% confluency. Cells were then infected with HCMV (multiplicity of infection = 1, 10^5^ PFU/ml) for 2 h, incubated for the indicated number of days post-infection, and then, washed, and refed with fresh ECM. To evaluate HCMV infectivity, immunofluorescence analysis was performed to examine infection rates of HCMV in HUVECs. After fixation with formaldehyde solution (4%), the cells were washed with PBS (phosphate-buffered saline) for three times, and subjected to indirect immunofluorescence analysis using anti-HCMV pp65 (Boster Corp., Wuhan, China), monoclonal antibodies, and fluorescein isothiocyanate-labeled sheep anti-rabbit IgG antibodies (dilution, 1:400; Boster Corp., Wuhan, China). The nuclei were stained with DAPI (4′6′-diamidino-2-phenylindole). The protein expression was observed under a fluorescence microscope.

### RNA extraction and gene expression microarray analysis

TRIzol reagent (provided by Invitrogen, Carlsbad, CA, America), chloroform, and isopropanol were used to extract total RNA from HCMV-infected or uninfected HUVECs according to the manufacturer manual. The extracted RNA was dissolved in 50 μL RNase-free H_2_O. The qualities and purities of the RNA preparations were determined by 1% agarose gel electrophoresis, and the quantities were determined by using an ND-1000 spectrophotometer (NanoDrop Technologies LLC, Wilmington, DE, America).

Microarray analysis was undertaken using RNA samples from HCMV-infected HUVECs, HUVECs treated with high glucose (HG; 4500 mg/L), and oxidized low-density lipoprotein (ox-LDL; 100 mg/L) for 48 h, as well as HCMV-infected HUVECs treated with HG and ox-LDL; uninfected cells were processed in parallel as a control. Microarray analysis was conducted by Beijing Biotech Co. Ltd. (Beijing, China). Four biological samples were added onto an Affymetrix PrimeView Human Gene Expression Array platform (Affymetrix Technologies, Inc., Santa Clara, CA, America), containing 49395 specific probes. RNA linear amplification was carried out according to the manufacturer manual. An Affymetrix Gene Chip Scanner 3000 (Affymetrix, CA, America) was employed to scan microarray slides after microarray hybridization. The raw data were converted logarithmically first, and the original data were standardized and analyzed using Affymetrix® GeneChip® Command Console® Software software. DEGs between the infected and control groups were recognized as genes with a FC (fold-change) > ±2 (*p* < 0.05). The full details of this microarray design have been deposited in the Gene Expression Omnibus public database (https://www.ncbi.nlm.nih.gov/geo) under platform accession number GSE 142762.

### Gene ontology (GO) and pathway enrichment analysis

Analysis of GO and Kyoto Encyclopedia of Genes and Genomes (KEGG) enrichment pathways was performed using the DAVID Bioinformatics Tool. Disease-related databases, including Online Mendelian Inheritance in Man, Genetic Association Database, the Human Disease Database (MalaCards), and genome-wide association studies (GWAS), were assessed as well.

### Validation of microarray results by quantitative reverse transcription polymerase chain reaction (RT-qPCR)

To confirm the results obtained by analysis of mRNA expression profiles, quantitative reverse transcription polymerase chain reaction was employed for determination of the expression level of dysregulated mRNAs. 3 µg total RNA which was extracted from each treatment group was reversely transcribed into cDNA (Tiangen Biotech, Shanghai, China), according to the manufacturer manual. In addition, RT-qPCR was carried out in a reaction system (25-μL) containing reverse primer, forward primer, SYBR Green/Fluorescein qPCR Master Mix and cDNA. The relative gene expression level was normalized using β-actin as the internal reference. 7300 Real-Time PCR system (provided by Applied Biosystems, Singapore) was employed. The SDS 2.0.1 software (provided by Applied Biosystems, Foster City, CA, USA) was used to calculate cycle threshold (Ct) values, which were normalized to β-Actin according to the 2^−ΔΔCt^ method. The sequences of the sense and antisense primers used for amplification are listed in Table 3.

**Table 3:** PCR primers of RT-qPCR.

Gene	Primer	Sequence	Amplicon size(bp)
ECE	Forward	5′TCCCGTCCTTGTCTACTCC3′	124
	Reverse	5′CAGGCACCATTCTACACACG3′	
RGS5	Forward	5′CACAAAGCGAGGCAGAGAAT3′	192
	Reverse	5′AAGATGGCTGAGAAGGCAAA3′	
SELE	Forward	5′TTCAGGACAGGCGAACTTG3′	133
	Reverse	5′GGGACAATGGACAGAAGAGG3′	
ATP1B1	Forward	5′GCCCAGTCCAAAATACTCCA3′	138
	Reverse	5′CCTAAGCCTCCCAAGAATGA3′	
PTGIS	Forward	5′ACTGGGGCTGTAATGTGGAA3′	144
	Reverse	5′GGCAGGTATGTCACCGTTCT3′	
HSD	Forward	5′GAAGAACTCGCCCACGAAC3′	125
	Reverse	5′GGGAGGAAGAGGAAGAGCAG3′	
ACE	Forward	5′CAGGGTGTGGTTGGCTATTT3′	114
	Reverse	5′CAGGTGGTGTGGAACGAGTA3′	
β-Actin	Forward	5′AATTCCATCATGAAGTGTGA3′	248
	Reverse	5′ACTCCTGCTTGCTGATCCAC3′	

Note. endothelin converting enzyme (ECE), regulator of G-protein signaling 5 (RGS5), selectin E (SELE), and ATPase, Na+ /K+ transporting, beta 1 polypeptide (ATP1B1), prostaglandin I2 (prostacyclin) synthase (PTGIS), 11-d hydroxysteroid dehydrogenase 2 (HSD), serum angiotensin-converting enzyme (ACE), β-Actin is housekeeping gene as a control.

### Establishment of a recombinant adenovirus for regulating overexpression of RGS5 (G-protein signaling 5)

We constructed recombinant adenoviruses which expressed RGS5 using the AdMEX adenoviral vector system. Both the shuttle vector (pHBdTrack-CMV) and backbone plasmid (pBHGlox [delta] E1, 3cre) for this system were provided by Shanghai Biotech Co. Ltd. (Shanghai, China). This pHBdTrack-CMV vector carries two separate CMV promoters which drive the expression of RGS5 and the GFP (green fluorescent protein). Targeted cells were inoculated into a 24-well plate (1 × 10^5^ cells/well). Cells were infected with the adenovirus carrying the target gene or an equal titer of control virus, and duplicate wells were infected for each group. After infection for 8 h, the virus was removed and replaced with a fresh medium. After 48 or 72 h, the level of fluorescence was measured by using a fluorescence microscope. Overexpression of RGS5 was confirmed by RT-qPCR as well.

### DNMT (nuclear DNA methyltransferase) activity assay

An EpiQuik Nuclear Extraction Kit (Aimeijie Biotech Inc., Wuhan, China) was used to extract nucleoprotein from HUVECs in different treatment groups. The extracted protein was then used for determination of the activity of DNMT, which was carried out using a DNMT activity assay kit (Aimeijie Biotech Inc., Wuhan, China), based on the manufacturer manual.

### DNA methylation analysis

Genomic DNA was extracted from HUVECs via a Blood and Tissue DNA Kit (provided by Qiagen Inc., Dusseldorf, Germany) based on the manufacturer’s protocols. The purity and concentration of the DNA were detected through measuring the absorbance at 280 and 260 nm. In total, 1.5 μg genomic DNA from each sample was treated with bisulfite using an EZ DNA Methylation-Gold Kit (provided by Zymo Research, Irvine, CA, America), in accordance with the manufacturer’s instructions. PCR primers were designed by using Epidesigner (Agena Bioscience, Inc., San Diego, CA, America). The methylation platform (CapitalBio Corp., Beijing, China), which included RNA base-specific cleavage and MALDI-TOF-MS (matrix-assisted laser desorption/ionization time-of-flight mass spectrometry), was employed for quantitative analysis on RGS5 methylation. The resulted methylation calls were analyzed by using EpiTyper 1.0 software (provided by Sequenom, San Diego, CA, America) to generate quantitative results for each CpG site or an aggregate of multiple CpG sites.

### CCK-8 (Cell Counting Kit-8) assay

To examine the effects of interactions between HCMV and environmental risk factors on the proliferation of HUVECs, proliferation of the cells was assayed by CCK-8 (Dojindo, Shanghai, China) based on the manufacturer manual. We seeded the cells in a 96-well plate (3 × 10^3^ cells/well), which were stained with CCK-8 dye (10 μL in 90 μL culture medium) at 24 h, 48 h, 72 h, and 96 h, respectively, for two hours at 37 °C. An ELx800 Microplate Reader (provided by BioTek Instruments Inc., Winooski, VT, America) set at 590 nm was employed to measure the absorbance. All procedures were repeated for three times.

### Western blot analysis

We extracted proteins from all experimental samples, which were separated by electrophoresis on 10% or 12% SDS-PAGE (sodium dodecyl sulfate-polyacrylamide gel electrophoresis). Proteins were then transferred onto PVDF (polyvinylidene difluoride) membranes. The membranes were incubated at four centigrade degree overnight with antibodies directed against the following proteins after blocking with nonfat dry milk (5%) dissolved in TBS-T for 2 h at room temperature: proliferating cell nuclear antigen (PCNA; dilution, 1:400; Boster Corp., Wuhan, China) and β-actin (dilution, 1:1000; Boster Corp., Wuhan, China). After washing, the membranes were incubated with horseradish peroxidase (HRP)-conjugated secondary antibodies (dilution, 1:25000; Boster Corp., Wuhan, China) for 2 h under room temperature. After TBS-T washing for three times, 10 min each time, an enhanced chemiluminescence system (Pierce Company, Waltham, MA, USA) was used to visualize the proteins. Eventually, expression levels were quantified by normalization to *β*-actin using Image J software (National Institutes of Health, Bethesda, MD, America).

### Statistical analysis

Mean ± standard deviation (SD) was used to describe the data. One- or two-way analysis of variance (ANOVA) was employed for intergroup comparison. The significant difference between groups was evaluated using two-tailed unpaired Student’s t-test. *P* < 0.05 indicated significant difference. SPSS 20.0 software (IBM Corp., Armonk, NY, USA) was employed for statistical analysis.

### Ethics approval and consent to participate

The study protocol was reviewed and approved by the Ethics Committee of First Affiliated Hospital of Medical College of Shihezi University (approval number: AF/SC-08/01.0) and performed in strict accordance with the rules ethics review of Biomedical research in human (2007) and the principles of the Declaration of Helsinki.

## Data availability

The analyzed data sets generated during the study are available from the corresponding author on reasonable request.

## Supplementary information

Dataset 1.

.Supplementary Information

## References

[1] Zhong MF (2011). Paradoxical effects of streptozotocin-induced diabetes on endothelial dysfunction in stroke-prone spontaneously hypertensive rats. The Journal of physiology.

[2] Lim SS (2012). A comparative risk assessment of burden of disease and injury attributable to 67 risk factors and risk factor clusters in 21 regions, 1990-2010: a systematic analysis for the Global Burden of Disease Study 2010. Lancet (London, England).

[3] Natekar A (2014). Elevated blood pressure: Our family’s fault? The genetics of essential hypertension. World journal of cardiology.

[4] Crough, T. & Khanna, R. Immunobiology of human cytomegalovirus: from bench to bedside. *Clinical microbiology reviews*, **22**, 76–98, Table of Contents (2009). 10.1128/CMR.00034-08 PMC2620639 19136435

[5] Reeves M, Sinclair J (2008). Aspects of human cytomegalovirus latency and reactivation. Current topics in microbiology and immunology.

[6] Stern-Ginossar N (2012). Decoding human cytomegalovirus. Science (New York, N.Y.).

[7] Li S (2011). Signature microRNA expression profile of essential hypertension and its novel link to human cytomegalovirus infection. Circulation.

[8] Haarala A (2012). Relation of high cytomegalovirus antibody titres to blood pressure and brachial artery flow-mediated dilation in young men: the Cardiovascular Risk in Young Finns Study. Clinical and experimental immunology.

[9] Li C, Samaranayake NR, Ong KL, Wong HK, Cheung BM (2012). Is human cytomegalovirus infection associated with hypertension? The United States National Health and Nutrition Examination Survey 1999-2002. PloS one.

[10] Vahdat K (2013). Association of pathogen burden and hypertension: the Persian Gulf Healthy Heart Study. American journal of hypertension.

[11] Tang N (2014). Human cytomegalovirus infection is associated with essential hypertension in Kazakh and Han Chinese populations. Medical science monitor: international medical journal of experimental and clinical research.

[12] Loenen WA, Bruggeman CA, Wiertz EJ (2001). Immune evasion by human cytomegalovirus: lessons in immunology and cell biology. Seminars in immunology.

[13] Streblow DN (1999). The human cytomegalovirus chemokine receptor US28 mediates vascular smooth muscle cell migration. Cell.

[14] Dharmashankar K, Widlansky ME (2010). Vascular endothelial function and hypertension: insights and directions. Current hypertension reports.

[15] Alcendor DJ, Charest AM, Zhu WQ, Vigil HE, Knobel SM (2012). Infection and upregulation of proinflammatory cytokines in human brain vascular pericytes by human cytomegalovirus. Journal of neuroinflammation.

[16] Jarvis MA, Nelson JA (2007). Human cytomegalovirus tropism for endothelial cells: not all endothelial cells are created equal. J Virol.

[17] Sinzger C (1995). Fibroblasts, epithelial cells, endothelial cells and smooth muscle cells are major targets of human cytomegalovirus infection in lung and gastrointestinal tissues. The Journal of general virology.

[18] Jarvis MA, Nelson JA (2002). Human cytomegalovirus persistence and latency in endothelial cells and macrophages. Current opinion in microbiology.

[19] Petrakopoulou P (2004). Cytomegalovirus infection in heart transplant recipients is associated with impaired endothelial function. Circulation.

[20] Zhao J (2018). Human cytomegalovirus infection-induced autophagy was associated with the biological behavioral changes of human umbilical vein endothelial cell (HUVEC). Biomedicine & pharmacotherapy = Biomedecine & pharmacotherapie.

[21] Shen K, Xu L, Chen D, Tang W, Huang Y (2018). Human cytomegalovirus-encoded miR-UL112 contributes to HCMV-mediated vascular diseases by inducing vascular endothelial cell dysfunction. Virus genes.

[22] Alikhani-Koopaei R, Fouladkou F, Frey FJ, Frey BM (2004). Epigenetic regulation of 11 beta-hydroxysteroid dehydrogenase type 2 expression. The Journal of clinical investigation.

[23] Bogdarina I, Welham S, King PJ, Burns SP, Clark AJ (2007). Epigenetic modification of the renin-angiotensin system in the fetal programming of hypertension. Circulation research.

[24] Frey FJ (2005). Methylation of CpG islands: potential relevance for hypertension and kidney diseases. Nephrology, dialysis, transplantation: official publication of the European Dialysis and Transplant Association - European Renal Association.

[25] Zhang D (2009). Epigenetics and the control of epithelial sodium channel expression in collecting duct. Kidney international.

[26] Feinberg AP, Ohlsson R, Henikoff S (2006). The epigenetic progenitor origin of human cancer. Nature reviews. Genetics.

[27] Gopalakrishnan S, Van Emburgh BO, Robertson KD (2008). DNA methylation in development and human disease. Mutation research.

[28] Krupanidhi S, Sedimbi SK, Sanjeevi CB (2008). Epigenetics and epigenetic mechanisms in disease with emphasis on autoimmune diseases. The Journal of the Association of Physicians of India.

[29] Friso S (2008). Epigenetic control of 11 beta-hydroxysteroid dehydrogenase 2 gene promoter is related to human hypertension. Atherosclerosis.

[30] Feng, Q. *et al*. Unexpected role of the human cytomegalovirus contribute to essential hypertension in the Kazakh Chinese population of Xinjiang. *Bioscience reports*, **38** (2018). 10.1042/BSR20171522 PMC6019381 29752343

[31] Esteki-Zadeh A (2012). Human cytomegalovirus infection is sensitive to the host cell DNA methylation state and alters global DNA methylation capacity. Epigenetics.

[32] Surekha RH (2007). Oxidative stress and total antioxidant status in myocardial infarction. Singapore medical journal.

[33] Bansal G, Druey KM, Xie Z (2007). R4 RGS proteins: regulation of G-protein signaling and beyond. Pharmacology & therapeutics.

[34] Adams LD, Geary RL, McManus B, Schwartz SM (2000). A comparison of aorta and vena cava medial message expression by cDNA array analysis identifies a set of 68 consistently differentially expressed genes, all in aortic media. Circulation research.

[35] Kirsch T, Wellner M, Luft FC, Haller H, Lippoldt A (2001). Altered gene expression in cerebral capillaries of stroke-prone spontaneously hypertensive rats. Brain research.

[36] Kach J, Sethakorn N, Dulin NO (2012). A finer tuning of G-protein signaling through regulated control of RGS proteins. American journal of physiology. Heart and circulatory physiology.

[37] Holobotovskyy V (2013). Regulator of G-protein signaling 5 controls blood pressure homeostasis and vessel wall remodeling. Circulation research.

[38] Li H (2010). Regulator of G protein signaling 5 protects against cardiac hypertrophy and fibrosis during biomechanical stress of pressure overload. Proceedings of the National Academy of Sciences of the United States of America.

[39] Chang PY, Qin L, Zhao P, Liu ZY (2015). Association of regulator of G protein signaling (RGS5) gene variants and essential hypertension in Mongolian and Han populations. Genetics and molecular research: GMR.

[40] Grayson TH (2007). Vascular microarray profiling in two models of hypertension identifies caveolin-1, Rgs2 and Rgs5 as antihypertensive targets. BMC genomics.

[41] Cho H (2008). Rgs5 targeting leads to chronic low blood pressure and a lean body habitus. Molecular and cellular biology.

[42] Hong K, Li M, Nourian Z, Meininger GA, Hill MA (2017). Angiotensin II Type 1 Receptor Mechanoactivation Involves RGS5 (Regulator of G Protein Signaling 5) in Skeletal Muscle Arteries: Impaired Trafficking of RGS5 in Hypertension. Hypertension (Dallas, Tex.: 1979).

[43] Arnold C (2018). Hypertension-evoked RhoA activity in vascular smooth muscle cells requires RGS5. FASEB journal: official publication of the Federation of American Societies for Experimental Biology.

[44] Romero M (2016). Vascular and Central Activation of Peroxisome Proliferator-Activated Receptor-beta Attenuates Angiotensin II-Induced Hypertension: Role of RGS-5. The Journal of pharmacology and experimental therapeutics.

[45] Billstrom Schroeder M, Christensen R, Worthen GS (2002). Human cytomegalovirus protects endothelial cells from apoptosis induced by growth factor withdrawal. Journal of clinical virology: the official publication of the Pan American Society for Clinical Virology.

[46] Cagliero E, Roth T, Roy S, Lorenzi M (1991). Characteristics and mechanisms of high-glucose-induced overexpression of basement membrane components in cultured human endothelial cells. Diabetes.

[47] Han J, Mandal AK, Hiebert LM (2005). Endothelial cell injury by high glucose and heparanase is prevented by insulin, heparin and basic fibroblast growth factor. Cardiovascular diabetology.

[48] Qiu J (2011). Coordination of Id1 and p53 activation by oxidized LDL regulates endothelial cell proliferation and migration. Annals of biomedical engineering.

[49] Li D, Mehta JL (2000). Upregulation of endothelial receptor for oxidized LDL (LOX-1) by oxidized LDL and implications in apoptosis of human coronary artery endothelial cells: evidence from use of antisense LOX-1 mRNA and chemical inhibitors. Arteriosclerosis, thrombosis, and vascular biology.

[50] Cominacini L (2000). Oxidized low density lipoprotein (ox-LDL) binding to ox-LDL receptor-1 in endothelial cells induces the activation of NF-kappaB through an increased production of intracellular reactive oxygen species. The Journal of biological chemistry.

[51] Chen J (2004). Role of caspases in Ox-LDL-induced apoptotic cascade in human coronary artery endothelial cells. Circulation research.

[52] Bentz, G. L. & Yurochko, A. D. Human CMV infection of endothelial cells induces an angiogenic response through viral binding to EGF receptor and beta1 and beta3 integrins. *Proceedings of the National Academy of Sciences of the United States of America* **105**, 5531-5536 (2008). 10.1073/pnas.0800037105 PMC2291133 18375753

[53] Batwa, S. A. *et al*. Prevalence of cytomegalovirus, and its effect on the expression of inducible and endothelial nitric oxide synthases in Fallopian tubes collected from women with and without ectopic pregnancy **35**, 103–110 (2016). 10.1007/s10096-015-2514-7 26563896

[54] Stenvinkel P (2007). Impact of inflammation on epigenetic DNA methylation - a novel risk factor for cardiovascular disease?. Journal of internal medicine.

[55] Yi-Deng J (2007). Folate and ApoE DNA methylation induced by homocysteine in human monocytes. DNA and cell biology.

[56] Zheng C (2015). Persistence of hAQP1 expression in human salivary gland cells following AdhAQP1 transduction is associated with a lack of methylation of hCMV promoter. Gene therapy.

[57] Feng, Q. *et al*. Unexpected role of the human cytomegalovirus contribute to essential hypertension in the Kazakh Chinese population of Xinjiang. *Bioscience reports* (2018). 10.1042/BSR20171522 PMC6019381 29752343

[58] Li J (2004). Regulator of G protein signaling 5 marks peripheral arterial smooth muscle cells and is downregulated in atherosclerotic plaque. Journal of vascular surgery.

[59] Xiao B, Zhang Y, Niu WQ, Gao PJ, Zhu DL (2009). Haplotype-based association of regulator of G-protein signaling 5 gene polymorphisms with essential hypertension and metabolic parameters in Chinese. Clinical chemistry and laboratory medicine.

[60] Ganss R (2015). Keeping the Balance Right: Regulator of G Protein Signaling 5 in Vascular Physiology and Pathology. Progress in molecular biology and translational science.

[61] Chen CH (2000). Oxidized low-density lipoproteins inhibit endothelial cell proliferation by suppressing basic fibroblast growth factor expression. Circulation.

[62] Chavakis E (2001). Oxidized LDL inhibits vascular endothelial growth factor-induced endothelial cell migration by an inhibitory effect on the Akt/endothelial nitric oxide synthase pathway. Circulation.

[63] Morss AS, Edelman ER (2007). Glucose modulates basement membrane fibroblast growth factor-2 via alterations in endothelial cell permeability. The Journal of biological chemistry.

[64] Reusser P (2002). Randomized multicenter trial of foscarnet versus ganciclovir for preemptive therapy of cytomegalovirus infection after allogeneic stem cell transplantation. Blood.

[65] Boeckh M, Geballe AP (2011). Cytomegalovirus: pathogen, paradigm, and puzzle. The Journal of clinical investigation.

[66] Prichard MN, Kern ER (2011). The search for new therapies for human cytomegalovirus infections. Virus Res.

