# Supplementary material for: Human cytomegalovirus promoting endothelial cell proliferation by targeting regulator of G-protein signaling 5 hypermethylation and downregulation
Source: Sci Rep. 2020 Feb 10;10:2252. doi: 10.1038/s41598-020-58680-6 (PMC7010708; doi:10.1038/s41598-020-58680-6)
Supplement: Supplementary file 2 — .Supplementary Information [file 41598_2020_58680_MOESM2_ESM.docx]

**SUPPLEMENTAL MATERIAL**

**Human cytomegalovirus promoting endothelial cell proliferation by targeting regulator of G-protein signaling 5 hypermethylation and downregulation**

Xiaoni Zhang^1,2†^, Na Tang ^1†^, Dongmei Xi^1^, Qian Feng ^1^, Yongmin Liu^1^, Lamei Wang^1^, Yan Tang^3^, Hua Zhong^1*^, Fang He^1*^

^1^Department of Pathophysiology/Key Laboratory of Education Ministry of Xinjiang Endemic and Ethnic Diseases, Medical College of Shihezi University, Shihezi, China.

^2^Second Department of Emergency and critical care medicine, the First Affiliated Hospital of Medical College of Shihezi University, Shihezi, China.

^3^Department of Geriatrics, the First Affiliated Hospital of Medical College of Shihezi University, Shihezi, China;

^†^These authors contribute equally to this work.

Supplementary Figure 4 SF


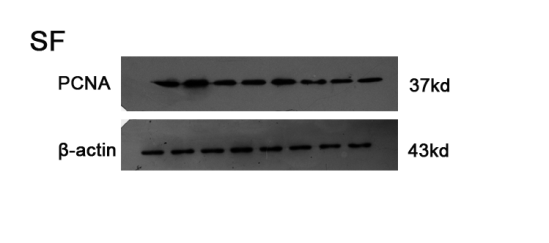


Supplementary Figure 5 SD


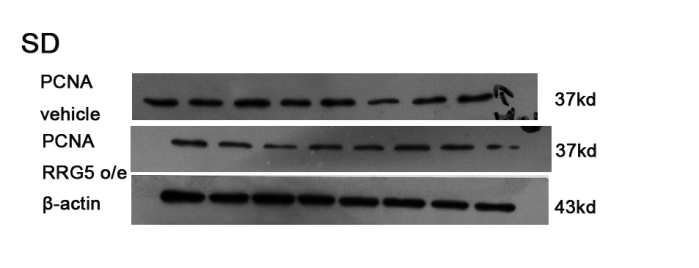


Supplemental Figure Legends

full-length blots_gels are presented in Supplementary Figure 4 SF：HCMV infection-induced EC proliferation was regulated by DNA methylation. Full-length blots_gels are presented in Supplementary Figure 4F.

full-length blots_gels are presented in Supplementary Figure 5 SD: Overexpression of RGS5 reversed EC proliferation triggered by HCMV infection. Full-length blots_gels are presented in Supplementary Figure 5D.
